# Catastrophic health expenditures and its inequality in elderly households with chronic disease patients in China

**DOI:** 10.1186/s12939-015-0134-6

**Published:** 2015-01-20

**Authors:** Zhonghua Wang, Xiangjun Li, Mingsheng Chen

**Affiliations:** School of Health Policy & Management, Nanjing Medical University, Nanjing, China; School of Economics and Management, Nanjing University of Traditional Medicine, Nanjing, China

**Keywords:** Catastrophic health expenditures, Inequality, Elderly households, China

## Abstract

**Background:**

Although numerous studies examine catastrophic health expenditures (CHE) worldwide, most focus on whole populations rather than specific vulnerable groups. This study analyzes the extent, associated factors and inequality of CHE in elderly household with chronic disease patients in China.

**Methods:**

Data were obtained from a nationally representative elderly household survey—the China Health and Retirement Longitudinal Study—that was conducted by the National School of Development of Peking University in 2011. An elderly household with chronic disease patients is defined by ≥ 1 chronic disease patient who is ≥ 45 years of age. CHE was measured according to the proportion of out-of-pocket health payments to non-food household expenditures. The associated factors of CHE were estimated using ordinary least square and logistic regression modeling. CHE inequality was measured according to the concentration index (CI) and its decomposition.

**Results:**

CHE incidence and intensity were relatively high among elderly households with chronic disease patients. The main associated factors of CHE include household size, having members > 65 years, having members with ≥ 2 chronic diseases, per capita income, and elderly household members demonstrating healthcare-seeking behaviors. Healthcare insurance did not significantly affect CHE risk. Disproportionate concentration of CHE was noted among elderly households, and poor elderly households demonstrated a higher probability of experiencing CHE. Factors such as household size, per capita income, having members > 65 years, and having members with ≥ 2 chronic diseases are major and positive contributors to CHE inequality. Some inpatient and outpatient services are negatively contributed to CHE inequality,suggesting that the unequal usage of such services reduces CHE inequality among elderly households with chronic disease patients.

**Conclusion:**

Policy efforts should focus on improving financial protection and relieving the economic burden of disease in elderly households. The government should increase income subsidies and optimize social health insurance programs, thereby reducing CHE and alleviating CHE inequality among elderly households in China.

## Introduction

The fundamental role of a healthcare system is to not only improve population health outcomes but also protect households from illness-associated financial catastrophe [1]. Health expenditures are considered “catastrophic” when they severely affect household living conditions, such as sending the household into poverty. Globally, 44 million households annually face catastrophic health expenditures (CHE), most of which are in low- and middle-income countries or countries that lack risk-sharing mechanisms [2,3].

Generally, two thresholds are widely used to define CHE: 1) out-of-pocket healthcare payments (OOP) that comprise ≥10% of total household expenditures [4-7]; and 2) out-of-pocket healthcare payments that comprise ≥40% of non-food household expenditures [8-11]. By deducting food expenses, the latter indicator can partly avoid measurement deviation that poor households which cannot afford to meet catastrophic payments are ignored [12]. The incidence of catastrophic healthcare payments dramatically differs between countries. Developed high-income countries, such as the United States, Germany, and Switzerland, have advanced social insurance or tax-funded health systems that protect households from catastrophic spending and reduce catastrophic incidence to < 1% (according to the 40% threshold). Meanwhile, in many developing low- or middle-income countries, such as Vietnam and Brazil, > 10% of households experience CHE (according to the 40% threshold) because of high rates of poverty and groups that are excluded from financial risk-protection mechanisms [13]. Like many developing countries, China is also facing high disease burden [14], OOP payments remain relatively high, and the overall incidence of catastrophic health expenses is about 13% (according to the 40% threshold) [15].

Chronic diseases often carry high economic burdens. Many studies indicate that households with members who have chronic conditions face higher financial risks than other households, and there is distributive income inequality in terms of CHE incidence and intensity [16-18]. Rahman et al. reported that in Bangladesh CHE incidence among households with members with chronic conditions is significantly greater than other households, and the financial risk of the lowest income households was about 3 times greater than the highest income households [19]. Another study in Bangalore also reported that CHE incidence among households with chronic disease patients is 16%, which notably exceeds the average level of the whole population and pro-poor CHE inequality exists among such households [20]. Meanwhile, several Chinese studies report that in rural China households with members who have chronic conditions have higher financial risks than other households [21-25], and the incidence of CHE is almost 1.5 times greater than the average level of whole population [26].

In recent years, the age of the Chinese population has increased. Chronic diseases occur more frequently in elderly people; hence, the prevalence of chronic diseases is rising with the rapidly aging population, which overburdens households with health expenditures and increases societal costs. In 2013, chronic conditions were responsible for 75% of all deaths, and the financial burden accounted for about 70% of the total economic disease burden [27]. Despite many studies examine CHE worldwide, most focus on whole populations rather than specific vulnerable groups. In China, the elderly are more sensitive to CHE, especially in rural areas, because of the high prevalence of chronic disease, low incomes, and the absence of social security mechanisms. This paper provides new knowledge about the extent, relevant factors and inequality of CHE in the elderly households with chronic disease patients in China. Our findings may contribute to improving and adjusting related healthcare policy, thereby further relieving the economic burden of disease.

## Methods and data

### Measuring CHE incidence and intensity

We measured CHE using the indicators reported by Wagstaff et al. [12]. We used non-food household expenditure instead of total household expenditure as the denominator in order to calculate CHE, and thereby partly avoid measurement deviations that are often ignored in poor households. The indicator, *E*, was calculated to determine whether CHE occurred:

1 $$ {E}_i=\left\{\begin{array}{ccc}\hfill 0\hfill & \hfill if\hfill & \hfill {T}_i/\left({x}_i-f(x)\right)<z\hfill \\ {}\hfill 1\hfill & \hfill if\hfill & \hfill {T}_i/\left({x}_i-f(x)\right)\ge z\hfill \end{array}\right. $$

Where T_*i*_ is the OOP payment for healthcare by household *i*; *x*_*i*_ is the total expenditures of household *i*; f(*x*) is the food expenditure of a household; and *z* is the given catastrophic threshold. The incidence and intensity of CHE were estimated as follows:

2 $$ Headcount=\raisebox{1ex}{$1$}\!\left/ \!\raisebox{-1ex}{$N$}\right.{\displaystyle {\sum}_{i=1}^N{E}_i} $$

3 $$ Overshoot=\raisebox{1ex}{$1$}\!\left/ \!\raisebox{-1ex}{$N$}\right.{\displaystyle {\sum}_{i=1}^N{E}_i}\left(\left({T}_i/\left({x}_i-f(x)\right)\right)-z\right) $$

4 $$ MPO=\raisebox{1ex}{$O$}\!\left/ \!\raisebox{-1ex}{$H$}\right. $$

In Formulas (2)-(4), *N* is the sample size. CHE incidence was estimated by performing a headcount. Headcount is the percentage of households whose OOP healthcare expenditures, as a fraction of non-food household expenditures, exceeded a particular threshold. CHE intensity was calculated using overshoot and mean positive overshoot (MPO). Overshoot measures the degree by which an average OOP health expenditure crosses the given catastrophic threshold of the entire sample, and MPO indicates the degree by which the average OOP health expenditure of a household exceeded the given threshold.

### CHE concentration index

Because of the diminishing marginal utility of income, the opportunity costs of healthcare spending by the poor will be greater than the rich [28]. In order to reflect this differential opportunity cost, we used a concentration index (CI) to measure CHE distribution in relation to income:

5 $$ CI=\raisebox{1ex}{$2$}\!\left/ \!\raisebox{-1ex}{$\mu $}\right.\operatorname{cov}\left(h,r\right) $$

Where *h* is the healthcare sector variable; *μ* is its mean; and *r* is the fractional rank of individuals in the distribution of income. A positive CI value indicates that CHE is greater among the rich, and a negative CI value indicates that the poor are more likely to experience CHE [29].

### CHE determinants and the decomposition of CI

Here, we define *x*_*k*_ as related factors to CHE, and the linear regression model of CHE and *x*_*k*_ can be written as follows:

6 $$ {Y}_i=\alpha +{\displaystyle {\sum}_k{\beta}_k}{x}_{ki}+{\varepsilon}_i $$

where *Y* denotes the CHE incidence or intensity. We used logistic regression modeling to estimate the related factors of incidence because incidence is a binary variable here. The concentration index can be decomposed into the contributions of individual factors to CHE inequality, in which each contribution is the product of the sensitivity of CHE with respect to that factor the degree of inequality in that factor [30]. Thus, the decomposition of CI can be calculated as follows:

7 $$ CI={\displaystyle {\sum}_k\left({\beta}_k{\overline{x}}_k/\overline{Y}\right)}C{I}_k+GC{I}_{\varepsilon }/\overline{Y} $$

Where $\overline{Y}$ is the mean CHE; ${\overline{x}}_k$ is the mean *x*_*k*_; *CI*_*k*_ is the concentration index for *x*_*k*_; and *GCI*_*ε*_ is the generalized concentration index of the error term *ε*.

### Data source

Data were obtained from a nationally representative elderly household survey, the China Health and Retirement Longitudinal Study (CHARLS), which is available online at: http://charls.ccer.edu.cn/en/page/data/2011-charls-wave1. This household survey, which included 150 counties in 28 provinces, aims to collect a high quality nationally representative sample of Chinese residents ages 45 and older to serve the needs of scientific research on the elderly [31]. Participants include person ≥ 45 years in any household and the individuals will be followed up every two years. The number of sampled households was 10,257, and the total number of participants was 17,708. Excluding missing data, 9530 sample households were eventually included. According to the CHARLS questionnaires, 14 chronic diseases were identified: hypertension, dyslipidemia (i.e., elevated low-density lipoprotein, triglycerides, and total cholesterol or low high-density lipoprotein), diabetes (i.e., high blood sugar), cancer or malignant tumor (except minor skin cancers), chronic lung disease (e.g., chronic bronchitis, emphysema), liver disease (except fatty liver, tumors, and cancer), heart disease (e.g., coronary heart disease, angina, congestive heart failure, other heart problems), stroke, kidney disease (except tumors or cancer), stomach and other digestive disease (except tumor or cancer), emotional and psychiatric problems, memory-related disease, arthritis or rheumatism, and asthma.

An elderly household with chronic disease patients is defined by ≥ 1 chronic disease patient who is ≥ 45 years of age. We identified 7695 such households, which accounted for 80.7% of all elderly households. Detailed descriptions of the variables are included in Table 1.

**Table 1:** **Description of variables**

**Variables**		**Description**	**Elderly household with chronic disease patients**	**Elderly household that did not report chronic disease**
			**Mean(S.D.)**	**Mean(S.D.)**
Household expenditure and OOP health expenditure				
OOP payment for health care		Household OOP medical expense over the last year (yuan RMB)	3227.07 (105.88)	1652.54 (207.06)
OOP payment of chronic disease patients		OOP medical expense of chronic disease patients over the last year (yuan RMB)	2513.83 (84.76)	-
Non-food household expenditure		Household Non-food expenditure over the last year (yuan RMB)	14921.84 (402.07)	12785.67 (596.89)
Household socioeconomic factors				
Marital status		=1 if married; =2 if divorced, widowed or never married.	1.1908 (0.0045)	1.3206 (0.0109)
Household size		=1 if living with children; =0 otherwise.	0.6570 (0.0054)	0.6990 (0.0107)
Receipt of economic assistance		=1 if receiving economic supports; =0 otherwise.	0.4104 (0.0056)	0.3206 (0.0109)
Provision of economic assistance		=1 if providing economic supports; =0 otherwise.	0.3605 (0.0055)	0.3495 (0.0111)
Providing care		=1 if spending time taking care of grandchildren or parents; =0 otherwise.	0.3940 (0.0056)	0.3128 (0.0110)
Household members > 65 years		=1 if having one or more members over 65 years of age; =0 otherwise.	0.3515 (0.0055)	0.2789 (0.0107)
Household members with ≥ 2 chronic diseases		=1 if any patient has two or more chronic diseases; =0 otherwise.	0.6398 (0.0055)	-
Per capita income		household income per capita over the last year(yuan RMB)	9653.96 (175.31)	10777.86 (405.67)
Healthcare insurance		=1if all patients have healthcare insurance; =0 otherwise.	0.9361 (0.2444)	0.9153 (0.2784)
Health-care seeking behaviors				
Outpatient service	Health care post or Village clinic	=1 if the elderly go to health care post or village clinic for treatment; =0 otherwise.	0.0955 (0.0034)	0.0447 (0.0048)
	First-level hospital	=1 if the elderly go to first-level hospital for treatment; =0 otherwise.	0.0496 (0.0025)	0.0224 (0.0035)
	Second-level hospital and above	=1 if the elderly go to second-level hospital or above for treatment; =0 otherwise.	0.0754 (0.0030)	0.0289 (0.0039)
Inpatient service	First-level hospital	=1 if the elderly go to first-level hospital for hospitalization; =0 otherwise.	0.0222 (0.0017)	0.0076 (0.0020)
	Second-level hospital and above	=1 if the elderly go to second-level hospital or above for hospitalization; =0 otherwise.	0.0611 (0.0027)	0.0311 (0.0041)

Note: Health care post or village clinic is a health facility that is primarily devoted to the care of outpatients in an urban or rural area; First-level hospitals refer to urban community health centers and township hospitals; second-level hospitals and above refer to general hospitals, specialized hospitals and Chinese medicine hospitals at county level and above.

Mean OOP healthcare payments by elderly households with chronic disease patients was 3227.07 yuan (RMB) (approximately US$ 509.89), which accounts for 21.6% of non-food household expenditures (Table 1). In contrast, mean OOP healthcare expenditure by other elderly households was only RMB 1652.54 (approximately US$ 262.31), which comprised 12.9% of non-food household expenditures. The OOP healthcare expenditures of chronic disease patients was RMB 2513.83 (approximately US$ 399.02), and its share of OOP health expenditure was about 77.9%.

## Results

### CHE incidence and intensity in elderly households

CHE incidence and intensity in elderly household are included in Table 2. As the threshold increased from 20% to 60% of non-food household expenditures, CHE incidence fell dramatically. CHE incidence and intensity were the greatest among rural elderly households with chronic disease patients at any given threshold (which is significantly different from urban elderly households), and CHE was significantly greater among elderly households with chronic disease patients in comparison with elderly households without chronic disease.

**Table 2:** **Incidence and intensity of CHE in elderly households**

		**Catastrophic threshold (share of non-food household expenditure)**				
		**20%**	**30%**	**40%**	**50%**	**60%**
Entire elderly households	Headcount	44.53%	33.62%	25.64%	19.59%	13.90%
	Overshoot	13.03%	9.14%	6.21%	3.95%	2.28%
	Mean positive overshoot	29.22%	27.20%	24.19%	20.14%	16.27%
Rural elderly households with chronic disease patients	Headcount	51.64%	39.68%	30.57%	23.46%	16.93%
	Overshoot	15.66%	11.10%	7.60%	4.88%	2.85%
	Mean positive overshoot	30.33%	27.98%	24.85%	20.82%	16.85%
Urban elderly households with chronic disease patients	Headcount	38.73%	28.44%	22.03%	17.23%	10.50%
	Overshoot	10.92%	7.57%	5.08%	3.10%	1.64%
	Mean positive overshoot	28.18%	26.63%	23.06%	17.97%	15.66%
Rural elderly households that did not report chronic disease	Headcount	29.10%	20.84%	13.54%	9.58%	7.19%
	Overshoot	7.52%	5.05%	3.33%	3.10%	1.34%
	Mean positive overshoot	25.85%	24.24%	24.57%	22.69%	18.61%
Urban elderly households that did not report chronic disease	Headcount	14.86%	9.87%	8.23%	5.12%	3.12%
	Overshoot	3.82%	2.60%	1.72%	1.07%	0.67%
	Mean positive overshoot	25.70%	26.28%	20.84%	20.88%	21.51%
Diff-1	Headcount	0.1200^**^	0.1042^**^	0.0808^**^	0.0579^**^	0.0520^**^
	(S.D.)	0.0136	0.0131	0.0123	0.0113	0.0100
	Overshoot	0.0446^**^	0.0332^**^	0.0237^**^	0.0166^**^	0.0109^**^
	(S.D.)	0.0058	0.0048	0.0038	0.0029	0.002
Diff-2	Headcount	−0.2214^**^	−0.1790^**^	−0.1521^**^	−0.1294^**^	−0.0934^**^
	(S.D.)	0.0131	0.0125	0.0115	0.0105	0.0092
	Overshoot	−0.0763^**^	−0.0561^**^	−0.0396^**^	−0.0254^**^	−0.0141^**^
	(S.D.)	0.0055	0.0045	0.0036	0.0027	0.0019

Note: Diff-1 is the difference of incidence and intensity between rural and urban elderly households with chronic disease patients; Diff-2 is the difference of incidence and intensity between elderly households with chronic disease patients and elderly households that did not report chronic disease; **implies significance at 0.01, and *indicates significance at 0.05.

Using a non-food expenditure threshold of 40% (a widely used threshold), the CHE incidence among all elderly households was 25.64%. Meanwhile, overshoot was 6.21%, which means elderly households on average spent 6.21% over the 40% catastrophic threshold. Elderly households that actually had experienced catastrophe at the 40% threshold spent an average of 24.19% (MPO) over this threshold. Thus, these households spent an average of 64.19% of all non-food expenditures on OOP medical expenses. Of all households sampled, rural elderly households with chronic disease patients demonstrated the greatest headcount (30.57%), overshoot (7.6%) and mean positive overshoot (24.85%), and urban elderly households with chronic disease patients demonstrated the second greatest headcount (22.03%) and overshoot (5.08%). As shown in Table 2, there were statistically significant differences in CHE between rural and urban households with chronic disease patients, households with chronic disease patients, and households without chronic disease.

### Determinants of CHE in elderly households with chronic disease patients

Table 3 shows the regression results of the related factors of CHE in elderly household with chronic disease patients at a non-food expenditure threshold of 40%. These results indicate a significant negative correlation between CHE incidence and intensity and household size, which means that CHE are more likely to occur in small elderly households. This effect was estimated to be greater in rural areas in comparison with urban areas. Providing economic assistance and the presence of household members > 65 years significantly increased the incidence and intensity of CHE in rural elderly households with chronic disease patients, but did not affect CHE risk in urban elderly households. Elderly households in which any patient had ≥ 2 chronic diseases were more likely to experience CHE, and the effect of having members with ≥ 2 chronic diseases on CHE incidence and intensity was estimated to be greater in urban areas than rural areas. Household per capita income was negatively correlated with CHE incidence and intensity. In other words, per capita income increased as CHE risk decreased. Healthcare insurance did not significantly affect the incidence and intensity of CHE in rural elderly households with chronic disease patients. CHE risk in rural elderly households was significantly higher when the elderly persons went to all categories of hospitals for outpatient and inpatient services, and this effect was associated with the hospital level. However, among urban elderly households, CHE was not statistically or significant affected when elderly patients went to hospitals at or above first-level for outpatient services or first-level hospitals for inpatient services. Only when urban elderly patients went to second-level hospitals and above for inpatient services was CHE incidence and intensity significantly increased.

**Table 3:** **Association between factors and CHE in elderly households with chronic disease patients (at a non-food expenditure threshold of 40%)**

		**Incidence of CHE**				**Intensity of CHE**			
		**Rural areas**		**Urban areas**		**Rural areas**		**Urban areas**	
		**Coefficient**	**S.D.**	**Coefficient**	**S.D.**	**Coefficient**	**S.D.**	**Coefficient**	**S.D.**
Marital status		0.1887	0.1638	0.2316	0.1747	−0.0004	0.0060	0.0188	0.0106
Household size		−0.3182^**^	0.0487	−0.4802^**^	0.0948	−0.0649^**^	0.0055	−0.0300^**^	0.0080
Receipt of economic assistance		0.0809	0.0462	−0.0718	0.1480	0.0075	0.0043	−0.0001	0.0116
Provision of economic assistance		0.1794^**^	0.0505	0.1081	0.1355	0.0190^**^	0.0047	0.0089	0.0091
Providing care		−0.0273	0.0485	−0.0024	0.0959	−0.0051	0.0043	−0.0054	0.0080
Household members > 65 years		0.1345^**^	0.0497	0.2252	0.1540	0.0126^*^	0.0052	0.0112	0.0096
Household members with ≥ 2 chronic diseases		0.3871^**^	0.0450	0.6050^**^	0.1255	0.0287^**^	0.0041	0.0339^**^	0.0074
Per capita income		−0.0153^**^	0.0038	−0.0366^**^	0.0131	−0.0057^**^	0.0014	−0.0070	0.0040
Healthcare insurance		0.2651	0.1557	−0.3086	0.2189	0.0069	0.0101	−0.0123	0.0110
Outpatient service	Health care post or village clinic	0.1927^**^	0.0667	−0.5182	0.3017	0.0194^**^	0.0070	−0.0295^*^	0.0127
	First-level hospital	0.3235^**^	0.0878	0.2535	0.2891	0.0234^*^	0.0098	0.0219	0.0377
	Second-level hospital and above	0.3537^**^	0.0905	0.1730	0.1577	0.0410^**^	0.0106	0.0174	0.0131
Inpatient service	First-level hospital	0.4539^**^	0.1083	−0.1315	0.6303	0.0676^**^	0.0163	0.0205	0.0419
	Second-level hospital and above	0.7031^**^	0.0937	0.9663^**^	0.2808	0.0931^**^	0.0118	0.0725^**^	0.0207
Constant		−0.1555	0.1434	−0.1850	0.4640	0.1337^**^	0.0158	0.0871^*^	0.0435

Note: **implies significance at 0.01; *indicates significance at 0.05.

### CHE inequality in elderly households

Given the differential opportunity costs of healthcare spending between the poor and rich, we measured the CHE distribution in relation to income. Table 4 shows the CI of CHE incidence and intensity in elderly households. As the threshold increased from 20% to 60% of non-food household expenditures, the CI of incidence and intensity were all negative: this means that poor elderly households are at higher risk of experiencing CHE. At the 40% threshold and above, we found that the absolute value of the CI (both incidence and intensity) of rural elderly households with chronic disease patients was greater than urban elderly households. Meanwhile, below the threshold of 40%, the absolute value of the CI for rural elderly households with chronic disease patients was smaller than urban elderly households.

**Table 4:** **Concentration indexes of CHE in elderly households**

		**Catastrophic threshold (share of non-food household expenditure)**				
		**20%**	**30%**	**40%**	**50%**	**60%**
Rural elderly households with chronic disease patients	Incidence	−0.0841	−0.1111	−0.1488	−0.1704	−0.1857
	Intensity	−0.1502	−0.1729	−0.1923	−0.2090	−0.2335
Urban elderly households with chronic disease patients	Incidence	−0.1246	−0.1438	−0.1433	−0.1284	−0.1522
	Intensity	−0.1433	−0.1465	−0.1482	−0.1559	−0.1867
Rural elderly households that did not report chronic disease	Incidence	−0.0981	−0.1448	−0.1955	−0.2049	−0.1905
	Intensity	−0.1718	−0.1951	−0.2098	−0.2128	−0.2274
Urban elderly households that did not report chronic disease	Incidence	−0.3362	−0.4117	−0.3956	−0.3833	−0.4021
	Intensity	−0.4205	−0.4418	−0.4595	−0.5069	−0.5765

### Decomposition of CHE inequality in elderly households with chronic disease patients

Tables 5 and 6 show the CI and relative contributions of each related factor of CHE inequality in elderly households with chronic disease patients at a non-food expenditure threshold of 40%. A positive contribution to socioeconomic inequality means that the relevant variable increases inequality, and vice versa. As shown in the fifth and the last column of Table 5, the majority of the observed inequalities in incidence in rural elderly households with chronic disease patients can be attributed to household size (72.00%), per capita income (21.22%), and having members > 65 years (13.84%), and provision of economic assistance (12.43%). The total contribution percentage is 108.84%, which means that 8.84% of the negative contribution to inequality in incidence is explained in the error term of the regression. In urban areas, the major positive contribution to inequality is mainly associated with household size (73.35%), having members with ≥ 2 chronic diseases (54.71%), per capita income (44.52%), and household members > 65 years (17.86%). The total contribution percentage is 98.88%, which means that 1.12% of the positive contribution to inequality in incidence is explained in the error term of the regression. The fifth and last column of Table 6 shows that the main positive contribution to inequality in intensity in rural elderly households with chronic disease patients is associated with household size (62.99%) and per capita income (28.10%), while the main contributors to urban elderly households with chronic disease patients are household size (67.14%), per capita income (38.26%) and household members with ≥ 2 chronic diseases (12.89%). The total contribution percentages are 107.35% and 96.76% respectively, which means that 7.35% of the negative contribution in rural area and 3.24% of the positive contribution in urban area are explained in the error term of the regression. Some inpatient and outpatient services are negatively contributed to CHE inequality, suggesting that the unequal usage of such services reduces CHE inequality in elderly households with chronic disease patients.

**Table 5:** **Decomposition of concentration index of incidence in elderly households with chronic disease patients (at a non-food expenditure threshold of 40%)**

		**Rural areas**				**Urban areas**			
		**Elasticity**	**Concentration index (CI)**	**Contribution to CI**	**Contribution to CI %**	**Elasticity**	**Concentration index (CI)**	**Contribution to CI**	**Contribution to CI %**
Marital status		0.6974	0.0397	0.0277	−18.61%	0.865	0.0591	0.0511	−35.66%
Household size		−0.7269	0.1474	−0.1071	72.00%	−1.2236	0.0859	−0.1051	73.35%
Receipt of economic assistance		0.1117	−0.0884	−0.0098	6.64%	−0.0936	−0.1343	0.0126	−8.79%
Provision of economic assistance		−0.2047	0.0906	−0.0185	12.43%	−0.1981	0.0741	−0.0147	10.26%
Providing care		−0.0349	0.0784	−0.0027	1.81%	−0.0047	0.073	−0.0003	0.21%
Household members > 65 years		0.1562	−0.132	−0.0206	13.84%	0.3505	−0.0729	−0.0256	17.86%
Household members with ≥ 2 chronic diseases		0.7874	−0.0161	−0.0127	8.53%	1.812	−0.0433	−0.0784	54.71%
Per capita income		−0.3505	0.0901	−0.0316	21.22%	−1.4713	0.0434	−0.0638	44.52%
Health insurance		0.8086	0.0048	0.0039	−2.61%	−1.2086	−0.0014	0.0017	−1.19%
Outpatient service	Health care post or Village clinic	0.0725	−0.0402	−0.0029	1.95%	−0.0566	−0.3169	0.0179	−12.49%
	First-level hospital	0.056	0.0364	0.002	−1.34%	0.0566	−0.0341	−0.0019	1.33%
	Second-level hospital and above	0.0722	0.0093	0.0007	−0.47%	0.1111	0.1978	0.022	−15.35%
Inpatient service	First-level hospital	0.0388	−0.143	−0.0055	3.70%	−0.0022	−0.1359	−0.0016	1.12%
	Second-level hospital and above	0.1232	−0.0368	−0.0045	3.02%	0.4483	0.099	0.0444	−30.98%
Total				−0.1619	108.84%			−0.1417	98.88%

**Table 6:** **Decomposition of concentration index of intensity in elderly households with chronic disease patients (at a non-food expenditure threshold of 40%)**

		**Rural areas**				**Urban areas**			
		**Elasticity**	**Concentration index (CI)**	**Contribution to CI**	**Contribution to CI %**	**Elasticity**	**Concentration index (CI)**	**Contribution to CI**	**Contribution to CI %**
Marital status		−0.0043	0.0397	−0.0002	0.09%	0.3049	0.0591	0.018	−12.15%
Household size		−0.5652	0.1474	−0.1211	62.99%	−1.1583	0.0859	−0.0995	67.14%
Receipt of economic assistance		0.0443	−0.0884	−0.0039	2.04%	−0.0008	−0.1343	0.0001	−0.07%
Provision of economic assistance		−0.0874	0.0906	−0.0079	4.12%	−0.0705	0.0741	−0.0052	3.51%
Providing care		−0.0264	0.0784	−0.0021	1.08%	−0.0454	0.073	−0.0033	2.23%
Household members > 65 years		0.0588	−0.132	−0.0078	4.04%	0.0757	−0.0729	−0.0055	3.71%
Household members with ≥ 2 chronic diseases		0.2352	−0.0161	−0.0038	1.97%	0.4407	−0.0433	−0.0191	12.89%
Per capita income		−0.5996	0.0901	−0.054	28.10%	−1.3067	0.0434	−0.0567	38.26%
Health insurance		0.0844	0.0048	0.0004	−0.21%	−0.2097	−0.0014	0.0003	−0.20%
Outpatient service	Health care post or Village clinic	0.0294	−0.0402	−0.0012	0.61%	−0.014	−0.3169	0.0044	−2.97%
	First-level hospital	0.0163	0.0364	0.0006	−0.31%	0.0212	−0.0341	−0.0007	0.47%
	Second-level hospital and above	0.0337	0.0093	0.0003	−0.16%	0.0484	0.1978	0.0096	−6.48%
Inpatient service	First-level hospital	0.0232	−0.143	−0.0033	1.73%	0.0015	−0.1359	−0.0002	0.13%
	Second-level hospital and above	0.0657	−0.0368	−0.0024	1.26%	0.1458	0.099	0.0144	−9.72%
Total				−0.2064	107.35%			−0.1434	96.76%

## Discussion

To the best of our knowledge, this study, which was conducted using a representative Chinese household survey, is the first to analyze the extent, relevant factors and inequality of CHE in elderly households with chronic disease patients. It is also one of the few studies to assess CHE in China [15,21-25].

Here, we found that 25.64% of elderly households demonstrate levels of health expenditure > 40% of their non-food expenditures and rural elderly households with chronic disease patients demonstrate the greatest incidence of CHE (30.57%), which are much higher than the overall CHE incidence assessed by another Chinese study (13.0% at the 40% threshold) [15]. This finding also demonstrates that the elderly’s risk tolerance for healthcare payments is actually lower than the average in China. CHE incidence and intensity in elderly households with chronic disease patients is significantly higher than elderly households that did not report chronic disease, which means that policy efforts need to focus on the elderly population with chronic disease in order to reduce household CHE and relieve the economic burden of chronic disease. Furthermore, more attention needs to be directed toward the rural elderly with chronic diseases because their households are at the greatest risk of experiencing CHE.

The factors associated with CHE incidence and intensity in elderly households with chronic disease patients include household size, providing economic assistance, having members > 65 years, having members with ≥ 2 chronic diseases, per capita income and health-care seeking behaviors. Specifically, large household size protects against CHE, which is more common in rural areas. Providing economic assistance and having members > 65 years increases the risk of CHE in rural elderly households with chronic disease, but does not affect urban elderly households; this indicates the rural–urban income gap and differences in family structure in China. The finding that healthcare insurances do not significantly affect CHE is similar to some existing literatures [25,32-34]. This demonstrates that healthcare insurance systems which mainly include some social health insurance programs in China actually have not reduced the risk of catastrophic spending and relieved financial burden of elderly population. The weak performance of social health insurance in financial protection maybe caused by the high prevalence of chronic diseases among elderly population and the corresponding medical expenditure pattern in policy design. Increasing compensation for chronic care of certain diseases should be a practicable way to improve the effectiveness and sustainability of health insurance system in China. The healthcare-seeking behaviors of the elderly affect CHE risk and demonstrated a huge discrepancy between rural and urban areas. In rural areas, CHE risk increased when elderly patients went to any type of hospital for outpatient or inpatient services. However, in urban areas, only when elderly patients went to second-level hospitals and above for inpatient services did CHE incidence and intensity significantly increase. Therefore, policies should be adjusted according to the differences between rural and urban areas in order to mitigate CHE risk in elderly households.

This study reveals the disproportionate concentration of CHE among poor elderly households and demonstrates that poor elderly households are at higher risk of experiencing CHE, similar to other published studies [18-20]. Hence, from a social welfare perspective, catastrophic payment problems are worse than it appears by simply looking at the fraction of the population that exceeds the threshold because this overlooks the fact that the poor tend to exceed this threshold [29]. In addition, we observed that CHE inequality increased more among rural elderly households with chronic disease patients than urban elderly households as the threshold increased, demonstrating that low-income rural elderly households with chronic disease patients are more likely to experience CHE. Factors such as household size, per capita income, having members > 65 years, and having members with ≥ 2 chronic diseases significantly affect the level of CHE and are major positive contributors to CHE inequality among elderly households with chronic disease patients. Hence, policies aimed at reducing CHE inequality must address the socioeconomic factors of healthcare outcomes among the elderly, such as implementing strategies that increase income among low-income elderly households, narrow the income gap, adjust the policy of healthcare insurance, and reduce the effects of socioeconomic status on CHE inequality. The unequal usage of some inpatient and outpatient services reduced CHE inequality among elderly households with chronic disease, thereby providing specific evidence that the government must adjust policies that affect the elderly population in order to decrease CHE inequality.

Our analysis shows that CHE severely affects the living conditions of elderly households with chronic disease patients in China. Therefore, policy efforts should further focus on enhancing financial protection and reducing CHE, especially among low-income rural elderly households with chronic disease patients. First, the government should gradually increase welfare subsidies or establish some kind of subsidiary fund that relieves the economic burden of disease in low-income rural elderly household with chronic disease patients. Second, the government should work to improve low-income elderly household’s ability of obtaining income, which will not only reduce CHE incidence and intensity but also decrease CHE inequality. Third, social health insurance programs that are organized and sponsored by government aim to protect its members against CHE, but according to our findings, this objective is actually not achieved in vulnerable groups. This is maybe caused by high medical costs, low effective reimbursement levels, and limited coverage [35]. Therefore, the government should further optimize and adjust these programs to reduce CHE and alleviate pro-poor CHE inequality among elderly households. Specifically, we need to reconsider benefit packages and redesign social healthcare insurance programs in order to further protect the elderly population with chronic disease. According to our findings, more attention should be paid to improving the reimbursement level of healthcare insurance programs to people > 65 years and elderly patients with ≥ 2 chronic diseases. In rural areas, this should further increase the reimbursement ratio of outpatient and inpatient expenditures to the elderly who receive treatment at every kind of hospitals. However, in urban areas, policy efforts should focus on improving the reimbursement ratio of inpatient spending to the elderly patients treated at county hospitals and above. In addition, medical financial assistance programs may need to be extended, especially to the poor and rural elderly, in order to supplement healthcare insurance and help further relieve the economic burden of disease.

Some limitations of our study must be acknowledged. We evaluated CHE incidence and intensity only in elderly households that actually presented for treatment and did not take into account elderly households that could not afford treatment. Many patients decide not to continue treatment because of insufficient resources. Thus, CHE incidence and intensity in elderly households might have been underestimated to some extent. In addition, our findings demonstrated that chronic disease is predictive of catastrophic health expenditure amongst households with elderly members but did not shed light on whether chronic or acute conditions are more likely to cause CHE. A lack of data and discussion regarding acute conditions is a limitation of the study.

## Conclusions

The findings revealed that CHE incidence and intensity were relatively high among elderly households with chronic disease patients. Furthermore, more attention needs to be directed toward the rural elderly with chronic diseases because their households are at the greatest risk of experiencing CHE. Some socioeconomic factors significantly affect CHE and are major positive contributors to CHE inequality among elderly households with chronic disease, which means that policies aimed at reducing CHE and its inequality must address the socioeconomic factors of healthcare outcomes among the elderly. Social health insurance programs have not reduced the risk of catastrophic spending and relieved financial burden of elderly population in China. Therefore, the government should further optimize and adjust these programs to further protect the elderly population with chronic disease.

### Ethics statement

The study was approved by the ethics committee of Nanjing Medical University and the ethical reviews board of Peking University. All participants provided written informed consent.

## References

[1] World Health Organization (2000). The World health report: 2000: Health systems: improving performance.

[2] Xu K, Evans DB, Carrin G, Aguilar-Rivera AM, Musgrove P (2007). Protecting households from catastrophic health spending. Health Aff (Millwood).

[3] Shahrawat R, Rao KD (2012). Insured yet vulnerable: out-of-pocket payments and India’s poor. Health Policy Plan.

[4] O’Donnell O, van Doorslaer E, Rannan-Eliya A, Somanathan CG (2005). Explaining the Incidence of Catastrophic Payments for Health Care: Comparative Evidence from Asia.EQUITAP Working Paper No.5.

[5] Galarraga O, Sosa-Rubi SG, Galarraga O, Salinas-Rodriguez A, Sesma-Vazquez S (2010). Health insurance for the poor: impact on catastrophic and out-of-pocket health expenditures in Mexico. Health Econ.

[6] Bredenkamp C, Mendola M, Gragnolati M (2011). Catastrophic and impoverishing effects of health expenditure: new evidence from the western Balkans. Health Policy Plan.

[7] Limwattananon S, Tangcharoensathien V, Prakongsai P (2007). Catastrophic and poverty impacts of health payments: results from national household surveys in Thailand. Bull World Health Organ.

[8] Xu K, Evans DE, Kawabate K, Zeramdini R, Klavus J, Murray CJL (2003). Household catastrophic health expenditure: a multicountry analysis.”. Lancet.

[9] Kavosi Z, Rashidian A, Pourreza A, Majdzadeh R (2012). Inequality in household catastrophic health care expenditure in a low income society of Iran. Health Policy Plan.

[10] Onwujekwe O, Hanson K, Uzochukwu B. Examining inequities in incidence of catastrophic health expenditures on different healthcare services and health facilities in Nigeria. PLoS One. 2012;7:e40811. 10.1371/journal.pone.0040811 PMC3397929 22815828

[11] Van Minh H, Phuong N, Saksena P, James CD, Xu K (2013). Financial burden of household out-of-pocket health expenditure in Viet Nam: findings from the national living standard survey 2002–2010. Soc Sci Med.

[12] Wagstaff A, van Doorslaer E (2003). Catastrophic and impoverishment in paying for health care: with application to Vietnam 1993–98. Health Econ.

[13] Xu K, Ravndal F, Evans DB, Carrin G (2009). Assessing the reliability of household expenditure data: results of the world health survey. Health Policy.

[14] Wagstaff A, Lindelow M (2008). Can insurance increase financial risk? The curious case of health insurance in China. J Health Econ.

[15] Wu QH, Li Y, Xu L, Hao YH (2012). Effect of health insurance on reduction of catastrophic health expenditure in China. Chinese J Health Policy.

[16] Onoka CA, Hanson K, Onwujekwe O, Uzochukwu B (2011). Examining catastrophic health expenditures at variable thresholds using household consumption expenditure diaries. Trop Med Int Health.

[17] Flores G, Krishnakumar J, O’Donnell O, van Doorslaer E (2008). Coping with health-care costs: implications for the measurement of catastrophic expenditures and poverty. Health Econ.

[18] Yardim MS, Cilingiroglu N, Yardim N (2010). Catastrophic health expenditure and impoverishment in Turkey. Health Policy.

[19] Rahman M, Gilmour S, Saito E, Sultana P, Shibuya K. Health-related financial catastrophe, inequality and chronic illness in Bangladesh. PLoS One. 2013;8:e56873. 10.1371/journal.pone.0056873 PMC3581555 23451102

[20] Bhojani U, Thriveni BS, Devadasan R, Munegowda CM, Devadasan N, Kolsteren P, et al. Out-of-pocket healthcare payments on chronic conditions impoverish urban poor in Bangalore. BMC Public Health. 2012;12:990. 10.1186/1471-2458-12-990 PMC3533578 23158475

[21] Cui Y, Liu JA, Wang Q, Yang L, Shi SH (2011). Analysis of catastrophic health payment among rural families with hypertension patients in poor region. Chinese Primary Health Care.

[22] Wan YY, Luo M, Lin Y, Zhang JY (2011). Analysis of catastrophic health payment in low-income rural household in China. Modern Preventive Medicine.

[23] Chen YY, Yin AT (2012). Research of catastrophic health expenditure determinants among rural inhabitants in Tengzhou city. Chinese Health Econ.

[24] Yan JE, Hao NN, Shi FM (2013). The changes and influencing factors on catastrophic health payment before and after new health care reform. Chinese J Health Policy.

[25] Jiang C, Ma J, Zhang X, Luo W. (2012) Measuring financial protection for health in families with chronic conditions in Rural China. BMC Public Health. Nov 16; 12:988. doi:10.1186/1471-2458-12-988. 10.1186/1471-2458-12-988 PMC3533936 23158260

[26] Chen YY, Yin AT, Zhao WJ, Chen YY, Yin AT, Zhao WJ, Li L, Wang P (2012). Research on the association of rural residents disease economic risk and disastrous health spending. Health Econ Res.

[27] Ministry of health. The chronic disease prevention plan of China 2012–2015. China: Ministry of Health; 2012. http://www.nhfpc.gov.cn/jkj/s5878/201205/a4e2a5d283e72c.shtml.

[28] Wagstaff A (2007). The economic consequences of health shocks: evidence from Vietnam. J Health Econ.

[29] O’Donnell O, van Doorslaer E, Wagstaff A, and Lindelow M. Analyzing health equity using household survey data: a guide to techniques and their implementation. World Bank Institute Learning Resources Series 202–210, World Bank Publications: Washington, DC USA; 2007.

[30] O’Donnell O, van Doorslaer E, Wagstaff A, and Lindelow M. Analyzing health equity using household survey data: a guide to techniques and their implementation. World Bank Institute Learning Resources Series 2007;159–164.

[31] National School of Development. China health and retirement longitudinal study. Peking University; 2012. http://charls.ccer.edu.cn/zh-CN.

[32] Liu H, Zhao Z. Impact of China’s urban resident basic medical insurance on health care utilization and expenditure. IZA Discussion Paper No. 6768. Bonn Germany, and Institute for The Study of labor: University of Bonn; 2012.

[33] Wagstaff A, Lindelow M, Gao J, Xu L, Qian JC (2009). Extending health insurance to the rural population: an impact evaluation of China’s New cooperative medical scheme. J Health Econ.

[34] Lei X, Lin S (2009). The new cooperative medical scheme in rural China: does more coverage mean more service and better health?. Health Econ.

[35] Zhang L, Cheng X, Tolhurst R, Tang SL, Liu XY (2010). How effectively can the new cooperative medical scheme reduce catastrophic health expenditure for the poor and non-poor in rural China. Trop Med Int Health.

